# Longitudinal Serum Protein Analysis of Women with a High Risk of Developing Breast Cancer Reveals Large Interpatient Versus Small Intrapatient Variations: First Results from the TESTBREAST Study

**DOI:** 10.3390/ijms232012399

**Published:** 2022-10-17

**Authors:** Sophie C. Hagenaars, Lennard J. M. Dekker, Bob Ravesteijn, Ronald L. P. van Vlierberghe, Fred P. H. T. M. Romijn, Linda Verhoeff, Arjen J. Witkamp, Karin E. Schenk, Kristien B. I. M. Keymeulen, Marian B. E. Menke-Pluijmers, Anneriet E. Dassen, Birgitta A. Kortmann, Jakob de Vries, Emiel J. T. Rutgers, Yuri E. M. van der Burgt, Elma Meershoek-Klein Kranenbarg, Christa M. Cobbaert, Theo M. Luider, Wilma E. Mesker, Rob A. E. M. Tollenaar

**Affiliations:** 1 Department of Surgery, Leiden University Medical Center, 2333 ZA Leiden, The Netherlands; 2 Department of Neurology, Neuro-Oncology Laboratory/Clinical and Cancer Proteomics, Erasmus University Medical Center, 3000 CA Rotterdam, The Netherlands; 3 Department of Clinical Chemistry and Laboratory Medicine, Leiden University Medical Center, 2333 ZA Leiden, The Netherlands; 4 Department of Surgery, Cancer Centre, University Medical Center Utrecht, 3584 CX Utrecht, The Netherlands; 5 Department of Surgery, Máxima Medical Center, 5504 DB Veldhoven, The Netherlands; 6 Department of Surgery, Maastricht University Medical Center, 6229 HX Maastricht, The Netherlands; 7 Department of Surgery, Albert Schweitzer Hospital, 3318 AT Dordrecht, The Netherlands; 8 Department of Surgery, Medisch Spectrum Twente, 7512 KZ Enschede, The Netherlands; 9 Department of Surgery, Spaarne Gasthuis, 2134 TM Hoofddorp, The Netherlands; 10 Department of Surgery, University Medical Center Groningen, 9713 GZ Groningen, The Netherlands; 11 Department of Surgery, Netherlands Cancer Institute, 1066 CX Amsterdam, The Netherlands

**Keywords:** breast cancer, proteomics, longitudinal analysis, serum, biomarkers, early detection, homeostasis, liquid biopsy, inter-intra patient variation, clustering

## Abstract

The prospective, multicenter TESTBREAST study was initiated with the aim of identifying a novel panel of blood-based protein biomarkers to enable early breast cancer detection for moderate-to-high-risk women. Serum samples were collected every (half) year up until diagnosis. Protein levels were longitudinally measured to determine intrapatient and interpatient variabilities. To this end, protein cluster patterns were evaluated to form a conceptual basis for further clinical analyses. Using a mass spectrometry-based bottom-up proteomics strategy, the protein abundance of 30 samples was analyzed: five sequential serum samples from six high-risk women; three who developed a breast malignancy (cases) and three who did not (controls). Serum samples were chromatographically fractionated and an in-depth serum proteome was acquired. Cluster analyses were applied to indicate differences between and within protein levels in serum samples of individuals. Statistical analyses were performed using ANOVA to select proteins with a high level of clustering. Cluster analyses on 30 serum samples revealed unique patterns of protein clustering for each patient, indicating a greater interpatient than intrapatient variability in protein levels of the longitudinally acquired samples. Moreover, the most distinctive proteins in the cluster analysis were identified. Strong clustering patterns within longitudinal intrapatient samples have demonstrated the importance of identifying small changes in protein levels for individuals over time. This underlines the significance of longitudinal serum measurements, that patients can serve as their own controls, and the relevance of the current study set-up for early detection. The TESTBREAST study will continue its pursuit toward establishing a protein panel for early breast cancer detection.

## 1. Introduction

Women who have an increased risk of developing breast cancer due to mutations in cancer susceptibility genes (i.e., *BRCA1, BRCA2*, *PALB2*, *ATM*, and *CHEK2*) or a familial predisposition undergo adapted screening programs [1] compared to women who are at population risk [2]. Although as a result of these programs, breast cancer is often detected at an early stage, the earlier onset of screening and higher number of screening moments leaves quite a burden on these women. Moreover, interval cancers can still occur between scheduled screening moments [3]. Hence, adding a regularly planned, relatively simple blood test to monitor biomarker levels that signal the onset of breast cancer at an early stage could be of added value in addition to the current imaging-based screening methods and can therefore improve breast cancer care.

Blood tests offer the possibility of a cheap and minimally invasive screening method with a high test performance, since a plethora of biomolecules are shed into peripheral fluids from the tumor site. These molecules are valuable sources of tumor-derived information. Various biomarkers can be selected as a target, among which circulating tumor cells (CTCs), microRNAs (miRNAs), and exosomes [4,5]. However, they have mostly demonstrated value with regard to survival and response to treatment [6,7,8] and are of limited use for diagnostic purposes. Therefore, the Trial Early Serum Test BREAST cancer (TESTBREAST) study was initiated with the goal of establishing a protein-based biomarker panel for the early detection of breast cancer in women with increased risk due to a familial or genetic predisposition [9,10]. Its particular set-up offers the opportunity for cases to serve as their own controls over time. The longitudinal collection of serum samples will potentially provide a unique protein biomarker signature, which can be used for the development of a blood test with high sensitivity and specificity for early cancer onset [11].

The TESTBREAST study makes use of a unique longitudinal study set-up for early detection [9], in contrast to many other protein-related biomarker projects, that follow a case–control design at the time of diagnosis [12]. The protein levels in the serum or plasma samples are rather stable over time, commonly rationalized by a phenomenon referred to as protein homeostasis, or proteostasis. A complex network of components dynamically balances cellular compositions with their functional requirements, where on the one hand, protein synthesis and folding are regulated and, on the other hand, misfolded and aggregated proteins are degraded [13]. In this context, it is hypothesized that secreted proteins or extracellular vesicles in blood provide accumulated evidence for the onset of a disease [14]. Often, more than one protein is associated with a disease, so an understanding of the dysregulated complex network over a longer time period follows from a multi-parameter approach.

Except for a relatively low number of routinely measured proteins, the precise intrapatient and interpatient variability of most protein levels over time is yet unknown. This is amongst others of importance with regard to determining the optimal method to select the distinctive proteins for early breast cancer detection. Therefore, we evaluated the protein levels of longitudinally acquired serum samples between and within the samples of different women who participate in the TESTBREAST study. These results will form a conceptual basis for further clinical analyses in a personalized way, aiming to compose a protein panel for the early detection of breast cancer.

## 2. Results

### 2.1. Patient Characteristics

In total, six moderate-to-high-risk women from the TESTBREAST study cohort were included in the analysis: three women who were diagnosed with breast cancer and three healthy controls (i.e., did not develop a breast malignancy during the study period or the 5-year follow-up period). Of all selected women, five longitudinal serum samples were available, leading to a total of 30 samples being analyzed. The maximum time between final sample and time of diagnosis was 40 days (range 0–40 days). Total protein levels were all within normal range (Table S1), and sample quality was sufficient (Figure S1). Age at diagnosis ranged between 36 and 55 years old. All tumors were of no special type (ductal carcinomas) and both hormone receptor negative and HER2-receptor negative (triple-negative breast cancer, TNBC). All cases had a high risk of developing breast cancer due to a *BRCA1* mutation; however, among the controls, there was one *BRCA2* mutation carrier and the others had an increased risk due to familial predisposition. Additional characteristics of the selected participating women are described in Table 1.

### 2.2. Clustering Analysis

**Figure 1:**
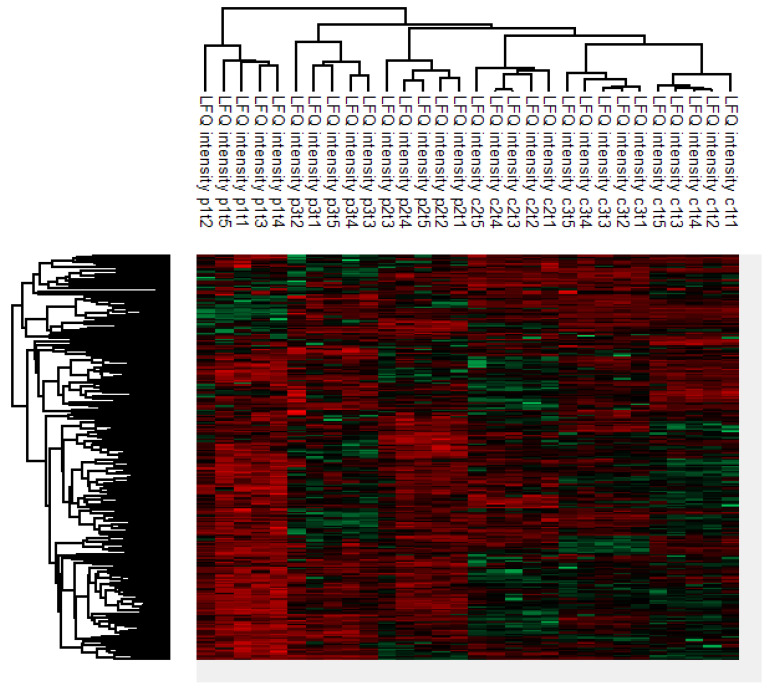
Heatmap combined with unsupervised hierarchical clustering based on z-score normalized LFQ intensities. Five longitudinally acquired serum samples of six individuals (three cases, three controls) were included. The high level of individual clustering indicates that intrapatient differences in protein levels ask for longitudinal sampling to detect subtle differences. c # = number of the control followed by the sequential time of blood draw; p # = number of the patient followed by the sequential time of blood draw.

As shown in Figure 1, a heatmap combined with unsupervised hierarchical clustering based on z-score normalized LFQ intensities was created to visualize grouping patterns in the dataset. A total of 764 proteins were identified, after which 267 proteins that passed the criterium of LFQ intensity data across all 30 samples were selected (i.e., the six individuals with five longitudinally acquired samples each) (Table S2). Every individual, i.e., both the cases and controls, formed a separate cluster including all five samples. Sequential samples within the cluster of an individual did not seem to form a cluster pattern in any specific order.

### 2.3. Intrapatient and Interpatient Variability in Protein Levels

**Figure 2:**
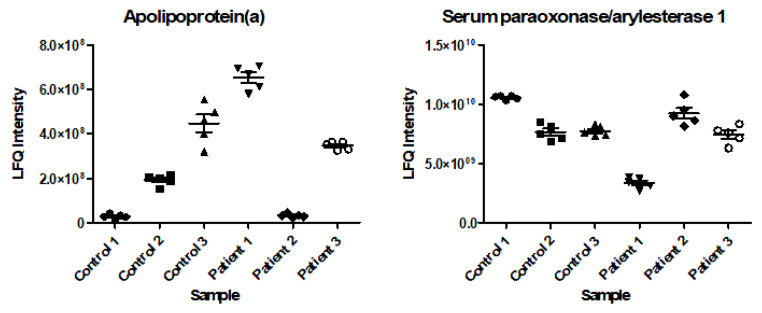
Two of the most distinctive proteins in the cluster analysis: apolipoprotein(a) (F-value 204.99; uniprot-accession code P08519) and serum paraoxonase/arylesterase 1 (F-value 91.62; uniprot-accession code P27169). LFQ intensity is presented as a linear value (mean and standard error of the mean); the individual dots represent the five longitudinally acquired samples for each patient and control.

**Figure 3:**
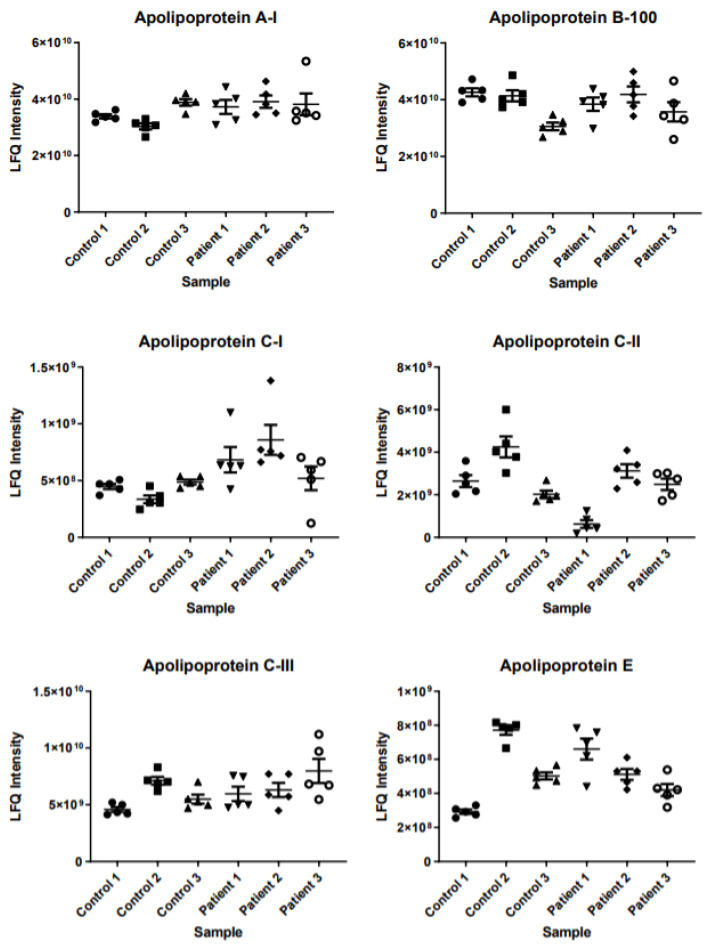
Clustering patterns for a selection of apolipoproteins. Reference values and uniprot-accession codes: apolipoprotein A-I (P02647) 1.2–1.8 g/L; apolipoprotein B-100 (P04114) 0.8–1.4 g/L; apolipoprotein C-I (P02654) 16–27 mg/L; apolipoprotein C-II (P02655) 19–55 mg/L; apolipoprotein C-III (P02656) 78–120 mg/L; apolipoprotein E (P02649) 18–43 mg/L [15].

Out of the 267 proteins analyzed in the ANOVA, 247 (92.5%) had an F-value higher than 2.62, ranging from 2.84 to 204.99. This means that for these 247 proteins, at least one of the individuals had a significantly different mean from the others at a 95% confidence interval. For the remaining 20 proteins (7.5%), the F-value was <2.62, ranging from 0.26 to 2.61. Therefore, for these 20 proteins, the means between individuals can be considered equal at a 95% confidence interval. Two of the proteins with high F-values are shown in Figure 2 (apolipoprotein(a) (P08519) with an F-value of 204.99 and serum paraoxonase (P27169) with an F-value of 91.62). Since one of the most distinctive proteins is an apolipoprotein, this encouraged us to also evaluate the levels of other apolipoproteins in detail. Figure 3 illustrates six additional apolipoproteins, together with its reference values [15], showing similar clustering patterns as described for apolipoprotein(a) and serum paraoxonase.

## 3. Discussion

This study presents the first results from the prospective, multicenter TESTBREAST study for the early detection of breast cancer in high-risk women. Using longitudinally acquired serum samples, both differences between patients and controls, as well as within each individual can be detected over time. Additionally, in this way, patients can serve as their own controls with regard to small changes in protein levels over time. The future aim is to develop a blood test based on a panel of distinctive proteins to detect breast cancer in the earliest stage possible or even before clinical diagnosis. To that end, protein level variations need to be considered. For most proteins, these variations are not known. Moreover, it is often assumed that protein patterns in healthy individuals are rather similar, rationalized as proteostasis. It is of great importance to understand these differences to allow a suitable selection of distinctive proteins for the development of the blood test for early detection.

Cluster analyses on a subset of TESTBREAST participants have revealed strong levels of clustering for longitudinally acquired samples of the same individual, indicating a larger interpatient than intrapatient difference in protein levels over time. This illustrates that the protein profile can be considered as “individual”, which has implications for the selection of interesting proteins for the development of the TESTBREAST blood test. The high level of clustering namely underlines the relevance of an individualized protein-based test, because if in the potential blood test all proteins would only be analyzed based on average cut-off levels (i.e., universally (ab)normal protein levels), small changes in the individual trend would be overlooked (i.e., individually (ab)normal protein levels). Therefore, the combination of proteins selected according to an average difference between cases and controls, together with proteins based on an individual change in trend starting 1–2 years before diagnosis, would be optimal for an individualized blood test. Hereby, an aberrant result following the blood test for one person could still be within normal values for another individual.

The TESTBREAST study was initiated in 2011 [9]. Since it requires great efforts to conduct a prospective, longitudinal study aimed at identifying a blood-based protein panel for the early detection of cancer [16,17], the TESTBREAST study has been including patients for 10 years. The cluster pattern described in this study underlines the need to organize the study in this way to enable tumor detection before clinical diagnosis or in the earliest stage possible and to longitudinally detect small differences in the protein levels of the individual patient. Additionally, it enables the determination of the baseline protein levels of a particular individual and, subsequently, follow-up of the personal protein levels over time to detect devious values. Moreover, a case–control study set-up based on single serum samples only obtained at the time of detection of the malignancy would not have been able to provide information on early detection but would merely present the difference between cases and controls at the time of diagnosis.

Proteomics research into breast cancer detection is often confined by a lack of identification and sequential validation studies of the proposed biomarkers [18], next to the minimal number of clinical, prospective trials that examine the added value of the protein biomarkers [19]. However, the potential of proteomics for diagnostic purposes is still acknowledged [20]. Using targeted proteomics in a longitudinally acquired cohort of 175 serum samples of matched cases and controls, Lee et al. have identified a protein classifier for the early detection of oropharyngeal squamous cell carcinoma [11]. Applying a comparable method to the one used in the TESTBREAST study, this study also underlines the importance of using longitudinal samples to identify biomarkers for cancer detection. *BRCA1* and *BRCA2* are well-known genetic risk factors for developing breast cancer that are determined once and do not provide any additional information in a person’s lifetime. Although these specific gene products (proteins) could not be measured in this study, a longitudinal protein or proteome analysis at an individual level reflects potential (subtle) changes in time, keeping in mind that the general protein levels are relatively stable (the earlier mentioned proteostasis).

This study has some limitations. First of all, there might be an influence of time of the measurement on the clustering pattern of the various proteins. However, one sample (sample 1, case 3; fractionated) was analyzed at a later point in time and still clustered in the same way. Secondly, all cases had a *BRCA1* mutation, while two controls had a high risk of developing breast cancer due to a familial predisposition and the other control was identified with a *BRCA2* mutation. Here, the cluster analysis indicated that there was no clear similarity between the *BRCA1* cases and the *BRCA2* control, compared to the controls without a known mutation. This is shown by the fact that the *BRCA2* control is positioned between the other controls with regard to its clustering pattern, therefore not more closely related to the cases, who also have *BRCA* gene mutations. Lastly, although the heatmap might give the illusion of being able to differentiate between cases and controls, based on their clustering pattern (Figure 1), this is unfortunately not possible. Additionally, it would be very time-consuming to distinguish women with early breast cancer from healthy controls based on this method; a simple blood test would be faster and more reliable.

## 4. Materials and Methods

### 4.1. Study Population

Women were eligible if they were between 25 and 75 years old at the time of inclusion, if they had a screening indication due to a familial or genetically increased breast cancer risk, or if their lifetime risk of breast cancer was determined to be higher than 15%. Finally, informed consent had to be obtained. Exclusion criteria were invasive breast cancer in the participant’s personal history or another malignancy in the last 10 years, apart from basal cell carcinoma.

In total, 1164 women were included in the study since the study initiation in 2011 and inclusions are ongoing. The number of cases is determined by the number of women who develop a breast carcinoma (event) during the study period. In the current study, a subset of three patients and three controls was selected for analysis, based on a minimum of five sequential serum samples per person. For the cases, the maximum period of time between time of diagnosis and the final sampling date was allowed to be one year. Controls were matched according to birth year, mutation status (i.e., *BRCA1/2*, *PALB2*, *ATM*, *CHEK2*, and other or no mutation) and serum sampling dates. For all controls, it was verified that no tumor was detected in the 5 years after the final blood sample was taken.

The TESTBREAST study is being conducted in accordance with the Declaration of Helsinki and approved by the Ethical Committee of the Leiden University Medical Center in agreement with the Dutch law for medical research involving human subjects. Moreover, local approval was obtained for all participating centers before the start of patient inclusion.

### 4.2. Serum Samples

Samples (550 μL and 4 × 500 μL in total) from moderate-to-high-risk women were collected every half year or year depending on the screening programs of the participating hospitals, up until diagnosis, the age of 75, or prophylactic mastectomy. Moreover, an extra blood sample was obtained in the case of an additional visit to the outpatient clinic because of a suspicious lesion or breast cancer diagnosis. The last sample was collected at the time of an event. If there was no blood sample obtained at the time of diagnosis, the serum sample closest to diagnosis was used for analyses. Importantly, blood draws had to occur before a biopsy was taken.

Serum was collected in BD Vacutainer SST II Advance tubes, after which the sample was processed by a lab technician in the laboratory. Newly obtained serum samples were centrifuged for 10 min at 1000 g, aliquoted, and stored in volumes of 550 μL and 4 × 500 μL at -80 degrees Celsius within 4 h after collection. Freeze/thaw cycles were avoided.

Samples used for the current study were obtained between 2008 and 2014. Before the start of the analyses, as a routine quality control, all samples were measured for sodium, potassium, chloride and total protein levels on a Cobas c502 analyzer (Roche Diagnostics, Mannheim, DE, USA) according to the manufacturer’s instructions to ensure optimal quality and representability.

### 4.3. Questionnaires

Next to blood samples, data were collected during the study by means of questionnaires to obtain information about the current health status of the participating women. Every study visit, study participants were asked to answer a total of ten questions about their current health status: the reason why they are regularly being seen at the outpatient clinic; the last time they had a biopsy or punction of the breast; any surgeries on the ovaries; menopause; use of exogeneous hormones; other complaints (e.g., infections or a fever); any underlying chronic diseases (e.g., COPD); use of medication; medical history of cancer; and smoking habit.

### 4.4. Data Management

Data coding, security and storage, including processes to promote data quality, were performed by an independent, qualified, and trained central data manager of the Clinical Research Center of the LUMC. Samples were administrated in Sample Navigator. 

ProMISe is the online data management system that was used for the TESTBREAST study. Inclusion and exclusion criteria were verified before a patient was registered in ProMISe. If a patient dropped out before the end of the study or if a participant developed a tumor, it was also noted in ProMISe.

### 4.5. Sample Preparation

First, each serum sample was thawed on ice and prepared according to mass spectrometry (MS)-based proteomics protocols including digestion with trypsin. Briefly, 90 μL of digestion buffer containing 50 mM triethylammonium bicarbonate buffer (TEAB) with 5% *v*/*v* acetonitrile (ACN) and 0.5% w/v sodium deoxycholate (SDC) was put into an empty Eppendorf tube per sample. Subsequently, 10 µL of the serum sample and 2.5 µL; 200 mM dithiothreitol (DTT) were added and shaken for 30 min at 56 °C. Next, 5.25 µL; 300 mM IAA was added and immediately stored in darkness for 30 min. Three µL of Trypsin Worthington (concentration: 10 µg/µL) was added, and the solution was shaken overnight at 37 °C. After that, 80 µL of the digestion solution was transported to a new empty Eppendorf tube and 80 µL; 1% trifluoroacetic acid (TFA), 5% ACN was added to stop the digesting process. This solution was centrifuged for 10 min at 3000g. Two aliquots were taken from the supernatant, 120 µL for fractionation and 10 µL for the addition of 90 µL; 0.1% TFA, 2% ACN in water to run a liquid chromatography-mass spectrometry (LC-MS) to check digestion.

After digestion, each sample was fractionated into 12 fractions using high-pH reverse-phase high-performance liquid chromatography (RP-HPLC) on an Ultimate 3000 LC system (Thermo Fisher Scientific, Germering, Germany) containing a C18 reversed phase column (Kinetex EVO, 2.1 mm × 150 mm, Phenomenex, Torrance, CA, USA), with 10 mM ammonium formate buffer pH 10 in water and 10 nM ammonium formate pH 10 in 80% ACN as mobile phases. For separation, a 28 min run was used. The run started with 4% organic mobile phase at a flow of 300 µL/min for 3 min, followed by a linear gradient from 4% to 38% at a flow of 450 µL/min for 9 min. Subsequently, the column was washed with 90% organic mobile phase at a flow of 450 µL/min for 6.1 min. Next, the system was equilibrated with 4% organic mobile phase for 9 min: first, for 6 min at a flow of 450 µL/min and, for the final 3 min, at a flow of 300 µL/min. Fraction collection occurred every 0.75 min between 6 min and 15 min.

The sample preparation process resulted in a total of 60 fractions per individual, based on 5 longitudinal serum samples that were each fractionated into 12 fractions. After drying, resuspending, and aliquoting, the samples were ready for MS analysis.

### 4.6. Mass Spectrometry

To analyse the proteins present in all serum samples, advanced liquid chromatography tandem mass spectrometry (LC-MS/MS) was used to obtain quantitative information about a large number of serum proteins. The fractionated serum samples were analyzed by a data-dependent acquisition (DDA) method. The Orbitrap Eclipse Tribrid MS system was used in combination with the nano-LC system (Ultimate 3000, Thermo Fisher Scientific, Germering, Germany) equipped with a reversed-phase column (PepMap C18, 75 μm internal diameter, 25 cm in length, 2 μm particle size, and 100 Å pore size). A linear 90 min gradient was used with 0.1% formic acid in water and 0.08% *v*/*v* formic acid in 80% *v*/*v* ACN as mobile phases, starting at 4% organic mobile phase, and increasing to 38% organic mobile phase at a flow of 300 µL/min.

The MS system was used with a FAIMS device (FAIMS Pro Interface, Thermo Fisher Scientific). The compensation voltages (CVs) of the device were set to −45, −60, −75, and −90, with a cycle time of 1 s. A static spray voltage of 2200 V was used, and the ion transfer tube temperature was set to 305 °C. For the parent scan, the resolving power of the Orbitrap was 120,000 and a scan range of 375–1500 *m*/*z* was applied. The automatic gain control (AGC) was 400,000, and the injection time was 50 ms. The isolation window was set to 1.6 *m*/*z*, a higher-energy collisional dissociation (HCD) was used with a normalized collision energy (NCE) of 30%, and the ion trap was operated in rapid scanning mode for the MS/MS scans with an AGC of 10,000 and an injection time of 35 ms. This untargeted method was used, since it allows for an extension of the depth in which the serum proteome can be investigated, and hereby, over 750 proteins in a serum sample can be identified.

### 4.7. Statistical Analysis

All raw data files were split into their separate CV (−45, −60, −75, and −90) spectra using Freestyle 1.6 (Thermo Fisher Scientific), resulting in 4 files per fraction and thus 48 unique files per sample.

Primary analysis of the mass spectra was conducted using MaxQuant (version 1.6.17.0) using each of the 30 samples as an “experiment” with fractions 1–48 for each individual split raw file. Standard settings were used unless indicated otherwise. The human subset of the uniprot database (downloaded 11 May 2020) was selected. Moreover, the label-free quantitation (LFQ) option was used. 

Perseus (version 1.6.14.0) and Microsoft Excel were used to further analyze and organize the MaxQuant output. The data were filtered by only including proteins with a valid LFQ intensity for all 30 samples and excluding any REVERSE (reference) proteins. Subsequently, a standard ANOVA was performed with a 95% confidence interval. The critical F-value was determined to be 2.62 (5, 24) degrees of freedom. Finally, a heatmap was composed using Perseus by logarithmically transforming the data, taking the z-score using standard settings and hierarchical clustering using standard settings.

## 5. Conclusions

In conclusion, using a subset of the longitudinally obtained serum samples of the TESTBREAST study cohort, a strong clustering pattern was identified, revealing a larger interpatient than intrapatient difference in protein levels. These results underline the unique, prospective, longitudinal serum sampling method used in the TESTBREAST study and the necessity of longitudinal sampling to be able to identify biomarkers for an individualized blood test for early breast cancer detection, which combines both individually and universally aberrant protein levels. Further analyses of the TESTBREAST study samples with regard to establishing and validating a protein panel for early detection using the entire TESTBREAST study cohort is currently ongoing, which will be followed by an external validation in collaboration with the NAF [9] and DENSE [21] studies.

## Figures and Tables

**Table 1:** Patient characteristics.

Patient		Birth Year	Year Tumor Diagnosis	Histology Tumor	Hormonal Status Tumor	Mutational Status	Medical History of Cancer	Menopause	Hormonal Use	Smoking	Medicine Use	Adnexal Surgery
Case	P1	1972	2014	Ductal (NST)	TNBC	BRCA1	No	Yes	Yes	Yes	No	Preventive adnex extirpation
Case	P2	1959	2014	Ductal (NST)	TNBC	BRCA1	No	Yes	No	No	Yes	Preventive adnex extirpation
Case	P3	1975	2011	Ductal (NST)	TNBC	BRCA1	No	Yes	No	No	Yes	Preventive adnex extirpation
Control	C1	1971	N/A	N/A	N/A	No, high risk	No	No	Yes	Sometimes	No	No
Control	C2	1960	N/A	N/A	N/A	No, high risk	Yes, ovarium carcinoma	Yes	Yes	No	No	Hysterosalpingo-oophorectomy
Control	C3	1969	N/A	N/A	N/A	BRCA2	No	No	No	No	No	No

Abbreviations: NST = no special type, TNBC = triple-negative breast cancer.

## Data Availability

The datasets analyzed during this study are available from the corresponding author upon reasonable request.

## Supplementary Materials

The following supporting information can be downloaded at https://www.mdpi.com/article/10.3390/ijms232012399/s1.

## List of Abbreviations

CTC	Circulating Tumor Cell
miRNA	microRNA
TESTBREAST	Trial Early Serum Test BREAST cancer
MS	Mass Spectrometry
TEAB	Triethylammonium Bicarbonate Buffer
ACN	Acetonitrile
SDC	Sodium Deoxycholate
DTT	Dithiothreitol
TFA	Trifluoroacetic Acid
LC-MS	Liquid Chromatography-Mass Spectrometry
RP-HPLC	Reverse-Phase High-Performance Liquid Chromatography
LC-MS/MS	Liquid Chromatography Tandem Mass Spectrometry
DDA	Data-Dependent Acquisition
CV	Compensation Voltages
AGC	Automatic Gain Control
HCD	Higher-Energy Collisional Dissociation
NCE	Normalized Collision Energy
LFQ	Label-Free Quantitation
TNBC	Triple-Negative Breast Cancer
