# Supplementary material for: Longitudinal Serum Protein Analysis of Women with a High Risk of Developing Breast Cancer Reveals Large Interpatient Versus Small Intrapatient Variations: First Results from the TESTBREAST Study
Source: Int J Mol Sci. 2022 Oct 17;23(20):12399. doi: 10.3390/ijms232012399 (PMC9604317; doi:10.3390/ijms232012399)
Supplement: Supplementary file 1 [file ijms-23-12399-s001.zip › ijms-1956654-supplementary.pdf]

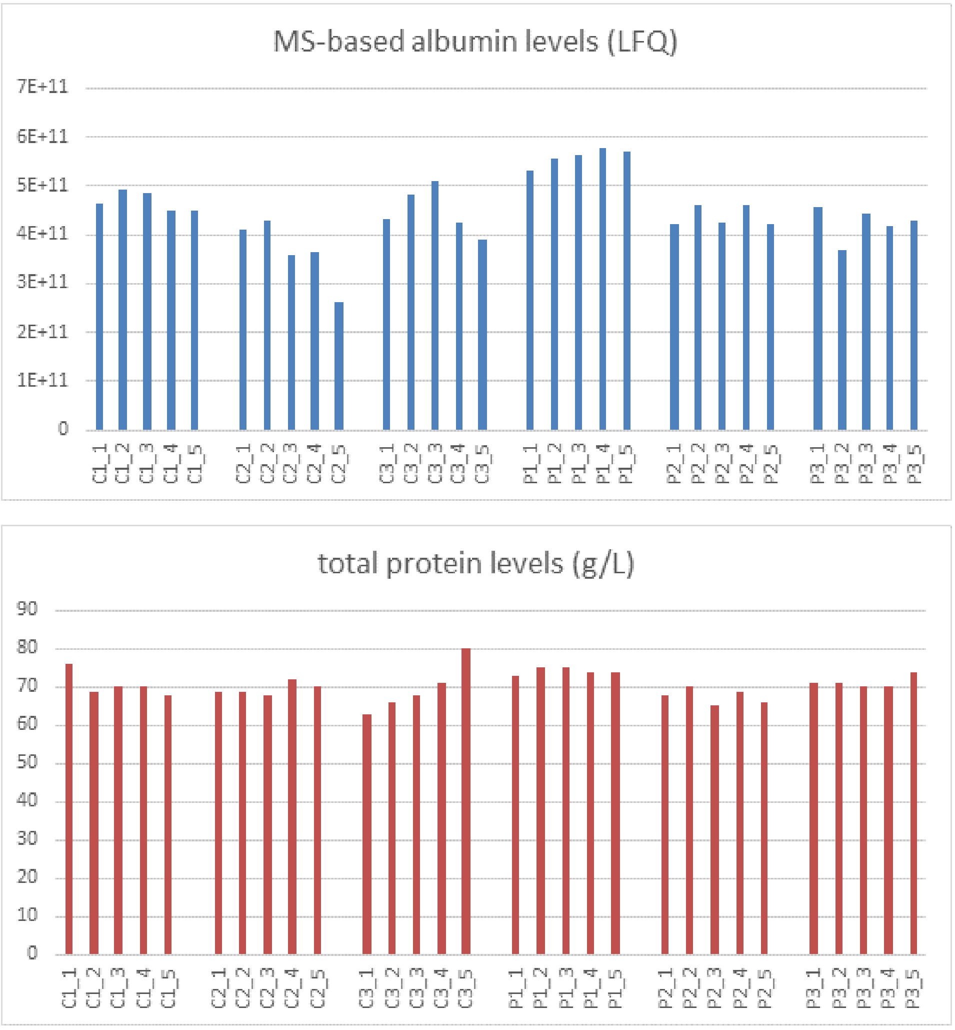

Figure S1. Serum albumin levels measured by mass spectrometer and the total protein levels as quantified by a clinical assay using a Cobas c502 analyzer (Roche Diagnostics, Mannheim, DE), functioning as a quality control of the samples. Five sequential serum timepoints of the three controls are shown on the left, cases on the right side.

Table S1. Sample control - Timepoint 5 (T5) is the serum sample closest to diagnosis.

**Case**	**Total proteins (range 66-87 g/L)**	**Control**	**Total proteins (range 66-87 g/L)**
Case 1 T1	76	Control 1 T1	73
Case 1 T2	69	Control 1 T2	75
Case 1 T3	70	Control 1 T3	75
Case 1 T4	70	Control 1 T4	74
Case 1 T5	68	Control 1 T5	74
Case 2 T1	69	Control 2 T1	68
Case 2 T2	69	Control 2 T2	70
Case 2 T3	68	Control 2 T3	65
Case 2 T4	72	Control 2 T4	69
Case 2 T5	70	Control 2 T5	66
Case 3 T1	63	Control 3 T1	71
Case 3 T2	66	Control 3 T2	71
Case 3 T3	68	Control 3 T3	70
Case 3 T4	71	Control 3 T4	70
Case 3 T5	80	Control 3 T5	74

Table S2. Overview of the 267 proteins that passed the criterium of LFQ intensity data, together with the majority protein IDs, protein names, gene names, IDs and F-values.

Majority protein ID	Protein name	Gene name	ID	F-value
P08519	Apolipoprotein(a)	LPA	330	204,9901818
P01859	Ig gamma-2 chain C region	IGHG2	200	140,4398941
P0DOX3	Immunoglobulin delta heavy chain		353	124,7547037
P26927	Hepatocyte growth factor-like protein;Hepatocyte growth factor-like protein alpha chain;Hepatocyte growth factor-like protein beta chain	MST1	467	97,51252789
P27169	Serum paraoxonase/arylesterase 1	PON1	470	91,62240576
P01861	Ig gamma-4 chain C region	IGHG4	202	88,84864448
P11226	Mannose-binding protein C	MBL2	380	76,71244479
P01721	Ig lambda chain V-VI region AR	IGLV6-57	184	71,14886699
P80748	Ig lambda chain V-III region LOI	IGLV3-21	566	63,45989495
P02776	Platelet factor 4;Platelet factor 4, short form	PF4	236	50,93543021
P01876	Ig alpha-1 chain C region	IGHA1	204	48,79517836
Q6YHK3	CD109 antigen	CD109	645	45,42294819
Q15485	Ficolin-2	FCN2	622	44,91808301
P0DP09; A0A0B4J2D9	Immunoglobulin kappa variable 1-13	IGKV1D-13	36	42,31795583
A0A075B6R2	Immunoglobulin heavy variable 4-4	IGHV4-4	11	41,3933345
A0A0B4J1U3	Immunoglobulin lambda variable 1-36	IGLV1-36	25	41,33594238
P13671	Complement component C6	C6	399	41,1401531
P00739	Haptoglobin-related protein	HPR	147	40,49682158
P01860	Ig gamma-3 chain C region	IGHG3	201	39,32285784
P36955	Pigment epithelium-derived factor	SERPINF1	499	38,91938274
Q92496	Complement factor H-related protein 4	CFHR4	685	37,59918944
P17936	Insulin-like growth factor-binding protein 3	IGFBP3	426	36,32463066
P02763	Alpha-1-acid glycoprotein 1	ORM1	231	35,95604107
Q6UXB8	Peptidase inhibitor 16	PI16	642	35,46243414
P0DOX2	Immunoglobulin alpha-2 heavy chain		352	34,41140409
Q6EMK4	Vasorin	VASN	636	34,01435553
P43251	Biotinidase	BTD	509	33,94966967
O43866	CD5 antigen-like	CD5L	113	33,82152976
Q14520	Hyaluronan-binding protein 2;Hyaluronan-binding protein 2 50 kDa heavy chain;Hyaluronan-binding protein 2 50 kDa heavy chain alternate form;Hyaluronan-binding protein 2 27 kDa light chain;Hyaluronan-binding protein 2 27 kDa light chain alternate form	HABP2	610	32,84851401

P00738	Haptoglobin;Haptoglobin alpha chain;Haptoglobin beta chain	HP	146	32,1597555
P01833	Polymeric immunoglobulin receptor;Secretory component	PIGR	198	31,41471026
O14786	Neuropilin-1	NRP1	105	31,40138875
P05546	Heparin cofactor 2	SERPIND1	283	31,18474822
P02786	Transferrin receptor protein 1;Transferrin receptor protein 1, serum form	TFRC	237	31,13622767
Q7Z7G0	Target of Nesh-SH3	ABI3BP	648	30,60667276
P01871	Ig mu chain C region	IGHM	203	30,11881174
P15169	Carboxypeptidase N catalytic chain	CPN1	411	29,04463113
P29622	Kallistatin	SERPINA4	477	28,26987802
P02788	Lactotransferrin;Lactoferricin-H;Kaliocin-1;Lactoferroxin-A;Lactoferroxin-B;Lactoferroxin-C	LTF	239	27,31388602
P04433; A0A0A0MRZ8	Ig kappa chain V-III region VG	IGKV3D-11	261	26,38595374
A0A075B6H9	Immunoglobulin lambda variable 4-69	IGLV4-69	0	26,18565375
P00748	Coagulation factor XII;Coagulation factor XIIa heavy chain;Beta-factor XIIa part 1;Coagulation factor XIIa light chain	F12	152	26,03164758
A0A0B4J1V0	Immunoglobulin heavy variable 3-15	IGHV3-15	27	25,92596001
P02649	Apolipoprotein E	APOE	211	25,88199745
P04278	Sex hormone-binding globulin	SHBG	258	25,54219418
A2NJV5	Immunoglobulin kappa variable 2-29	IGKV A18	62	24,48567541
P06276	Cholinesterase	BCHE	285	24,43548937
Q76LX8	A disintegrin and metalloproteinase with thrombospondin motifs 13	ADAMTS13	646	23,87113983
P07357	Complement component C8 alpha chain	C8A	305	23,76148776
P14625	Endoplasmin	HSP90B1	406	22,89309747
Q9Y5Y7	Lymphatic vessel endothelial hyaluronic acid receptor 1	LYVE1	759	22,87658836
Q08380	Galectin-3-binding protein	LGALS3BP	584	22,73382986
P07358	Complement component C8 beta chain	C8B	306	22,19881404
P08697	Alpha-2-antiplasmin	SERPINF2	336	22,1167544
P01023	Alpha-2-macroglobulin	A2M	160	21,92780343
P0DJI8	Serum amyloid A-1 protein;Amyloid protein A;Serum amyloid protein A(2-104);Serum amyloid protein A(3-104);Serum amyloid protein A(2-103);Serum amyloid protein A(2-102);Serum amyloid protein A(4-101)	SAA1	348	21,01332697
P0DOX5; P01857	Ig gamma-1 chain C region	IGHG1	355	21,00149317
Q96IY4	Carboxypeptidase B2	CPB2	693	20,82373336
P05156	Complement factor I;Complement factor I heavy chain;Complement factor I light chain	CFI	275	20,57320276

Q04756	Hepatocyte growth factor activator;Hepatocyte growth factor activator short chain;Hepatocyte growth factor activator long chain	HGFAC	579	20,40510236
O75882	Attractin	ATRN	127	20,3903698
P01703	Ig lambda chain V-I region NEWM	IGLV1-40	179	20,1963928
P02655	Apolipoprotein C-II;Proapolipoprotein C-II	APOC2	214	20,15880602
P02787	Serotransferrin	TF	238	20,0483842
Q9BXR6	Complement factor H-related protein 5	CFHR5	707	19,67076742
P01619	Ig kappa chain V-III region B6	IGKV3-20	174	19,63534486
P00746	Complement factor D	CFD	150	19,52807981
P33151	Cadherin-5	CDH5	488	19,06578892
P19652	Alpha-1-acid glycoprotein 2	ORM2	435	18,95625521
P04275	von Willebrand factor;von Willebrand antigen 2	VWF	257	18,3404499
Q92954	Proteoglycan 4;Proteoglycan 4 C-terminal part	PRG4	690	18,09535217
Q03591	Complement factor H-related protein 1	CFHR1	577	17,68876171
P01714	Ig lambda chain V-III region SH	IGLV3-19	181	17,42203857
Q9UGM5	Fetuin-B	FETUB	739	17,35599574
P01042	Kininogen-1;Kininogen-1 heavy chain;T-kinin;Bradykinin;Lysyl-bradykinin;Kininogen-1 light chain;Low molecular weight growth-promoting factor	KNG1	165	17,28548424
P11021	78 kDa glucose-regulated protein	HSPA5	375	17,12774992
Q92820	Gamma-glutamyl hydrolase	GGH	687	16,91123585
Q12913	Receptor-type tyrosine-protein phosphatase eta	PTPRJ	592	16,52302718
O14791	Apolipoprotein L1	APOL1	106	16,48824193
P10643	Complement component C7	C7	368	15,94615629
P08571	Monocyte differentiation antigen CD14;Monocyte differentiation antigen CD14, urinary form;Monocyte differentiation antigen CD14, membrane-bound form	CD14	332	15,93222346
P04196	Histidine-rich glycoprotein	HRG	253	15,62937288
Q9UHG3	Prenylcysteine oxidase 1	PCYOX1	740	15,39158446
Q06033	Inter-alpha-trypsin inhibitor heavy chain H3	ITIH3	580	15,33582345
P22891	Vitamin K-dependent protein Z	PROZ	450	15,31074079
P07225	Vitamin K-dependent protein S	PROS1	298	15,23747764
P00747	Plasminogen;Plasmin heavy chain A;Activation peptide;Angiostatin;Plasmin heavy chain A, short form;Plasmin light chain B	PLG	151	14,88465516
P00734	Prothrombin;Activation peptide fragment 1;Activation peptide fragment 2;Thrombin light chain;Thrombin heavy chain	F2	144	14,87214195

P00736	Complement C1r subcomponent;Complement C1r subcomponent heavy chain;Complement C1r subcomponent light chain	C1R	145	14,68690751
P35443	Thrombospondin-4	THBS4	491	14,67267973
P23142	Fibulin-1	FBLN1	453	14,64295405
P08185	Corticosteroid-binding globulin	SERPINA6	320	14,61448394
Q07954	Prolow-density lipoprotein receptor-related protein 1;Low-density lipoprotein receptor-related protein 1 85 kDa subunit;Low-density lipoprotein receptor-related protein 1 515 kDa subunit;Low-density lipoprotein receptor-related protein 1 intracellular domain	LRP1	583	14,50390371
P0C0L5	Complement C4-B;Complement C4 beta chain;Complement C4-B alpha chain;C4a anaphylatoxin;C4b-B;C4d-B;Complement C4 gamma chain	C4B	347	14,46212259
Q9Y6R7	IgGFc-binding protein	FCGBP	761	14,43720695
P12259	Coagulation factor V;Coagulation factor V heavy chain;Coagulation factor V light chain	F5	390	14,17584805
P08709	Coagulation factor VII;Factor VII light chain;Factor VII heavy chain	F7	337	14,078676
P01824	Ig heavy chain V-II region WAH	IGHV4-39	197	13,98442496
P01701	Ig lambda chain V-I region NEW	IGLV1-51	178	13,8405282
Q15582	Transforming growth factor-beta-induced protein ig-h3	TGFBI	623	13,77803898
P01594; P01593	Ig kappa chain V-I region AU;Ig kappa chain V-I region AG		169	13,70475301
P27918	Properdin	CFP	473	13,67685267
P11597	Cholesteryl ester transfer protein	CETP	385	13,56392574
Q13093	Platelet-activating factor acetylhydrolase	PLA2G7	593	13,51306496
P06396	Gelsolin	GSN	288	13,39775077
P05452	Tetranectin	CLEC3B	281	13,3191491
P20742	Pregnancy zone protein	PZP	439	13,26009203
P14780	Matrix metalloproteinase-9;67 kDa matrix metalloproteinase-9;82 kDa matrix metalloproteinase-9	MMP9	408	13,21782773
P01591	Immunoglobulin J chain	IGJ	168	12,97744208
A0A0B4J1V2	Immunoglobulin heavy variable 2-26	IGHV2-26	29	12,86973141
P02765	Alpha-2-HS-glycoprotein;Alpha-2-HS-glycoprotein chain A;Alpha-2-HS-glycoprotein chain B	AHSG	232	12,77593877
P02751	Fibronectin;Anastellin;Ugl-Y1;Ugl-Y2;Ugl-Y3	FN1	228	12,55287988
A0A075B6S6	Immunoglobulin kappa variable 2D-30	IGKV2D-30	14	12,5467899
Q15113	Procollagen C-endopeptidase enhancer 1	PCOLCE	617	12,53978791
P02760	Protein AMBP;Alpha-1-microglobulin;Inter-alpha-trypsin inhibitor light chain;Trypstatin	AMBP	230	12,32978372

Q15063; CON__Q2KJC7	Periostin	POSTN	616	12,28381375
P07195	L-lactate dehydrogenase B chain	LDHB	297	12,26423427
P02750	Leucine-rich alpha-2-glycoprotein	LRG1	227	12,22915252
P49747	Cartilage oligomeric matrix protein	COMP	516	12,14141617
P12111	Collagen alpha-3(VI) chain	COL6A3	389	11,97514762
P05155	Plasma protease C1 inhibitor	SERPING1	274	11,73910191
A0A0C4DH36	Probable non-functional immunoglobulin heavy variable 3-38	IGHV3-38	47	11,6671925
P15144	Aminopeptidase N	ANPEP	409	11,59839464
A0A0B4J1X5	Immunoglobulin heavy variable 3-74	IGHV3-74	32	11,59304579
P54108	Cysteine-rich secretory protein 3	CRISP3	523	11,50763282
O00187	Mannan-binding lectin serine protease 2;Mannan-binding lectin serine protease 2 A chain;Mannan-binding lectin serine protease 2 B chain	MASP2	100	11,47117968
P18065	Insulin-like growth factor-binding protein 2	IGFBP2	428	11,29127617
P0DOX7	Immunoglobulin kappa light chain		357	11,16022737
P51884	Lumican	LUM	521	11,1354501
Q9NQ79	Cartilage acidic protein 1	CRTAC1	727	11,07967191
P03952	Plasma kallikrein;Plasma kallikrein heavy chain;Plasma kallikrein light chain	KLKB1	243	10,92920834
Q15166	Serum paraoxonase/lactonase 3	PON3	619	10,91688781
P07360	Complement component C8 gamma chain	C8G	308	10,9019407
P40197	Platelet glycoprotein V	GP5	505	10,86766753
P0DOY2	Immunoglobulin lambda constant 2	IGLC2	359	10,85476035
P01601	Ig kappa chain V-I region HK101	IGKV1D-16	172	10,83377976
P08603	Complement factor H	CFH	333	10,76818983
P01008	Antithrombin-III	SERPINC1	156	10,76049755
P01009	Alpha-1-antitrypsin;Short peptide from AAT	SERPINA1	157	10,72521689
P02775	Platelet basic protein;Connective tissue-activating peptide III;TC-2;Connective tissue-activating peptide III(1-81);Beta-thromboglobulin;Neutrophil-activating peptide 2(74);Neutrophil-activating peptide 2(73);Neutrophil-activating peptide 2;TC-1;Neutrophil-activating peptide 2(1-66);Neutrophil-activating peptide 2(1-63)	PPBP	235	10,71365159
Q16706	Alpha-mannosidase 2	MAN2A1	630	10,47827361
A0A075B6K5	Immunoglobulin lambda variable 3-9	IGLV3-9	8	10,42227854
P02768; CON__P02768-1	Serum albumin	ALB	74	10,1273927
P0DOX8	Immunoglobulin lambda-1 light chain		358	10,03837644

P04430	Ig kappa chain V-I region BAN	IGKV1-16	260	10,00047652
P01780	Ig heavy chain V-III region JON	IGHV3-7	193	9,851458873
P01599	Ig kappa chain V-I region Gal	IGKV1-17	171	9,784995375
A0A0C4DH68; A0A075B6R9	Immunoglobulin kappa variable 2-24	IGKV2-24; IGKV2D-24	12	9,748542539
P43121	Cell surface glycoprotein MUC18	MCAM	508	9,743876608
P02743	Serum amyloid P-component;Serum amyloid P-component(1-203)	APCS	221	9,680021388
P05160	Coagulation factor XIII B chain	F13B	276	9,652143361
O00533	Neural cell adhesion molecule L1-like protein;Processed neural cell adhesion molecule L1-like protein	CHL1	102	9,467642542
P22792	Carboxypeptidase N subunit 2	CPN2	449	9,438943822
P35858	Insulin-like growth factor-binding protein complex acid labile subunit	IGFALS	497	9,40003882
P01031	Complement C5;Complement C5 beta chain;Complement C5 alpha chain;C5a anaphylatoxin;Complement C5 alpha chain	C5	162	9,290600181
P00742	Coagulation factor X;Factor X light chain;Factor X heavy chain;Activated factor Xa heavy chain	F10	149	9,249220079
Q13201	Multimerin-1;Platelet glycoprotein Ia*;155 kDa platelet multimerin	MMRN1	595	9,111050315
P02671	Fibrinogen alpha chain;Fibrinopeptide A;Fibrinogen alpha chain	FGA	216	9,086666533
P24821	Tenascin	TNC	462	9,029117284
P09871	Complement C1s subcomponent;Complement C1s subcomponent heavy chain;Complement C1s subcomponent light chain	C1S	344	9,006521927
P01011	Alpha-1-antichymotrypsin;Alpha-1-antichymotrypsin His-Pro-less	SERPINA3	158	8,938512701
P06727	Apolipoprotein A-IV	APOA4	291	8,853129768
A0A075B6P5; P01615	Ig kappa chain V-II region FR	IGKV2D-28	9	8,834006015
P0C0L4	Complement C4-A;Complement C4 beta chain;Complement C4-A alpha chain;C4a anaphylatoxin;C4b-A;C4d-A;Complement C4 gamma chain	C4A	346	8,800630192
O75636	Ficolin-3	FCN3	125	8,752889447
P02753	Retinol-binding protein 4;Plasma retinol-binding protein(1-182);Plasma retinol-binding protein(1-181);Plasma retinol-binding protein(1-179);Plasma retinol-binding protein(1-176)	RBP4	229	8,739944542
P20851	C4b-binding protein beta chain	C4BPB	441	8,733343271
P01742	Ig heavy chain V-I region EU	IGHV1-69	185	8,518221012
Q9Y4L1	Hypoxia up-regulated protein 1	HYOU1	757	8,344583081
A0A0B4J1Y9	Immunoglobulin heavy variable 3-72	IGHV3-72	35	8,030105539

P00751	Complement factor B;Complement factor B Ba fragment;Complement factor B Bb fragment	CFB	153	8,001646567
P00450	Ceruloplasmin	CP	138	7,999513597
P07996; CON__Q28194	Thrombospondin-1	THBS1	317	7,753679164
P07359	Platelet glycoprotein Ib alpha chain;Glycocalicin	GP1BA	307	7,751611325
P60709; P63261	Actin, cytoplasmic 1;Actin, cytoplasmic 1, N-terminally processed;Actin, cytoplasmic 2;Actin, cytoplasmic 2, N-terminally processed	ACTB; ACTG1	540	7,652225374
P54289	Voltage-dependent calcium channel subunit alpha-2/delta-1;Voltage-dependent calcium channel subunit alpha-2-1;Voltage-dependent calcium channel subunit delta-1	CACNA2D 1	524	7,498042657
Q8NBP7	Proprotein convertase subtilisin/kexin type 9	PCSK9	669	7,476159134
P01700	Ig lambda chain V-I region HA	IGLV1-47	177	7,378994672
P02748	Complement component C9;Complement component C9a;Complement component C9b	C9	225	7,378052347
O95445	Apolipoprotein M	APOM	131	7,36046524
A0A075B6K4	Immunoglobulin lambda variable 3-10	IGLV3-10	7	7,352392319
P05090	Apolipoprotein D	APOD	268	7,340181447
Q6UX71	Plexin domain-containing protein 2	PLXDC2	641	7,254507351
P04003	C4b-binding protein alpha chain	C4BPA	244	7,221840397
P08195	4F2 cell-surface antigen heavy chain	SLC3A2	321	6,997363649
Q9NZP8	Complement C1r subcomponent-like protein	C1RL	731	6,984814624
Q16610	Extracellular matrix protein 1	ECM1	628	6,967108833
P98160	Basement membrane-specific heparan sulfate proteoglycan core protein;Endorepellin;LG3 peptide	HSPG2	567	6,940668369
Q12860	Contactin-1	CNTN1	588	6,892273198
P48740	Mannan-binding lectin serine protease 1;Mannan-binding lectin serine protease 1 heavy chain;Mannan-binding lectin serine protease 1 light chain	MASP1	514	6,860012373
P61769	Beta-2-microglobulin;Beta-2-microglobulin form pI 5.3	B2M	545	6,677691994
P04070	Vitamin K-dependent protein C;Vitamin K-dependent protein C light chain;Vitamin K-dependent protein C heavy chain;Activation peptide	PROC	248	6,568638828
P10909	Clusterin;Clusterin beta chain;Clusterin alpha chain	CLU	374	6,518380748
P03951	Coagulation factor XI;Coagulation factor XIa heavy chain;Coagulation factor XIa light chain	F11	242	6,495424064
P01024	Complement C3;Complement C3 beta chain;C3-beta-c;Complement C3 alpha chain;C3a anaphylatoxin;Acylation stimulating protein;Complement C3b alpha chain;Complement C3c alpha chain fragment 1;Complement C3dg fragment;Complement C3g fragment;Complement C3d	C3	161	6,458678065

	fragment;Complement C3f fragment;Complement C3c alpha chain fragment 2			
P01602	Ig kappa chain V-I region HK102	IGKV1-5	173	6,349863645
P02679	Fibrinogen gamma chain	FGG	218	6,298520971
P02774	Vitamin D-binding protein	GC	234	6,292861724
P02675	Fibrinogen beta chain;Fibrinopeptide B;Fibrinogen beta chain	FGB	217	6,149522775
P02749	Beta-2-glycoprotein 1	APOH	226	6,126900363
P80108	Phosphatidylinositol-glycan-specific phospholipase D	GPLD1	563	6,108926239
A0A0B4J1U7	Immunoglobulin heavy variable 6-1	IGHV6-1	26	6,106281015
P05556	Integrin beta-1	ITGB1	284	6,085348648
P43652	Afamin	AFM	510	5,927231415
Q96PD5	N-acetylmuramoyl-L-alanine amidase	PGLYRP2	696	5,922691846
P03950	Angiogenin	ANG	241	5,883617596
P02747	Complement C1q subcomponent subunit C	C1QC	224	5,756444726
O00391	Sulfhydryl oxidase 1	QSOX1	101	5,725906214
Q9H4A9	Dipeptidase 2	DPEP2	713	5,69427735
A0A0C4DH67; A0A0C4DH69	Immunoglobulin kappa variable 1-8	IGKV1-8; IGKV1-9	52	5,665059377
Q96KN2	Beta-Ala-His dipeptidase	CNDP1	694	5,635980289
P25311	Zinc-alpha-2-glycoprotein	AZGP1	463	5,560267468
A0A0A0MS15	Immunoglobulin heavy variable 3-49	IGHV3-49	23	5,537640312
P02746	Complement C1q subcomponent subunit B	C1QB	223	5,476354219
P09486	SPARC	SPARC	342	5,456623826
P19320	Vascular cell adhesion protein 1	VCAM1	434	5,414576576
P00488	Coagulation factor XIII A chain	F13A1	140	5,339223375
A0A0C4DH72	Immunoglobulin kappa variable 1-6	IGKV1-6	53	5,276496174
P13796	Plastin-2	LCP1	401	5,226856952
P06681	Complement C2;Complement C2b fragment;Complement C2a fragment	C2	289	5,182896142
Q13103	Secreted phosphoprotein 24	SPP2	594	5,090331615
Q14624	Inter-alpha-trypsin inhibitor heavy chain H4;70 kDa inter-alpha-trypsin inhibitor heavy chain H4;35 kDa inter-alpha-trypsin inhibitor heavy chain H4	ITIH4	613	5,062499162
P19823	Inter-alpha-trypsin inhibitor heavy chain H2	ITIH2	436	4,845485256
P05543	Thyroxine-binding globulin	SERPINA7	282	4,754456855
P01019	Angiotensinogen;Angiotensin-1;Angiotensin-2;Angiotensin-3;Angiotensin-4;Angiotensin 1-9;Angiotensin 1-7;Angiotensin 1-5;Angiotensin 1-4	AGT	159	4,729817666

P04004	Vitronectin;Vitronectin V65 subunit;Vitronectin V10 subunit;Somatomedin-B	VTN	245	4,602305247
P01624	Ig kappa chain V-III region POM	IGKV3-15	175	4,577904542
P02656	Apolipoprotein C-III	APOC3	215	4,457365195
P02652	Apolipoprotein A-II;Proapolipoprotein A-II;Truncated apolipoprotein A-II	APOA2	212	4,280061845
P04114	Apolipoprotein B-100;Apolipoprotein B-48	APOB	250	4,260675258
P02790	Hemopexin	HPX	240	4,18808978
P22352	Glutathione peroxidase 3	GPX3	446	4,080072782
P39060	Collagen alpha-1(XVIII) chain;Endostatin	COL18A1	503	3,973038792
P02654	Apolipoprotein C-I;Truncated apolipoprotein C-I	APOC1	213	3,910669604
P01834	Ig kappa chain C region	IGKC	199	3,813767724
P33908	Mannosyl-oligosaccharide 1,2-alpha-mannosidase IA	MAN1A1	489	3,800173301
P35542	Serum amyloid A-4 protein	SAA4	493	3,624006683
P04406	Glyceraldehyde-3-phosphate dehydrogenase	GAPDH	259	3,592176464
P05154	Plasma serine protease inhibitor	SERPINA5	273	3,49112152
P08253	72 kDa type IV collagenase;PEX	MMP2	324	3,454069121
P15151	Poliovirus receptor	PVR	410	3,40733249
P22105	Tenascin-X	TNXB	445	3,25029076
Q13822	Ectonucleotide pyrophosphatase/phosphodiesterase family member 2	ENPP2	606	3,226108683
P02647	Apolipoprotein A-I;Proapolipoprotein A-I;Truncated apolipoprotein A-I	APOA1	210	3,132242057
P01033	Metalloproteinase inhibitor 1	TIMP1	163	3,051476479
P02766	Transthyretin	TTR	233	2,943858711
P06312	Ig kappa chain V-IV region	IGKV4-1	287	2,846413107
P14543	Nidogen-1	NID1	404	2,613375946
P00915	Carbonic anhydrase 1	CA1	154	2,594549656
P02745	Complement C1q subcomponent subunit A	C1QA	222	2,592587678
Q14766	Latent-transforming growth factor beta-binding protein 1	LTBP1	615	2,510159406
P55058	Phospholipid transfer protein	PLTP	528	2,285980033
P19827	Inter-alpha-trypsin inhibitor heavy chain H1	ITIH1	437	2,241609444
P04217	Alpha-1B-glycoprotein	A1BG	255	2,158069858
Q13790	Apolipoprotein F	APOF	605	2,008189366
Q6UWP8	Suprabasin	SBSN	640	1,9723215
Q7Z7M0	Multiple epidermal growth factor-like domains protein 8	MEGF8	649	1,849044014
Q9UK55	Protein Z-dependent protease inhibitor	SERPINA10	743	1,758411904

P04180	Phosphatidylcholine-sterol acyltransferase	LCAT	252	1,730695395
Q12805	EGF-containing fibulin-like extracellular matrix protein 1	EFEMP1	587	1,724635423
P00740	Coagulation factor IX;Coagulation factor IXa light chain;Coagulation factor IXa heavy chain	F9	148	1,71002366
P68871	Hemoglobin subunit beta;LVV-hemorphin-7;Spinorphin	HBB	558	1,624860109
Q6UY14	ADAMTS-like protein 4	ADAMTSL 4	643	1,343220363
P18428	Lipopolysaccharide-binding protein	LBP	430	1,240522784
P69905	Hemoglobin subunit alpha	HBA1	560	0,841222331
A0A0C4DH38	Immunoglobulin heavy variable 5-51	IGHV5-51	48	0,838427785
P11717	Cation-independent mannose-6-phosphate receptor	IGF2R	387	0,264799369
